# Ubiquitination of basal VEGFR2 regulates signal transduction and endothelial function

**DOI:** 10.1242/bio.027896

**Published:** 2017-08-10

**Authors:** Gina A. Smith, Gareth W. Fearnley, Izma Abdul-Zani, Stephen B. Wheatcroft, Darren C. Tomlinson, Michael A. Harrison, Sreenivasan Ponnambalam

**Affiliations:** 1 Endothelial Cell Biology Unit, School of Molecular & Cellular Biology, University of Leeds, Leeds LS2 9JT, UK; 2 Leeds Institute of Cardiovascular & Metabolic Medicine, Faculty of Medicine & Health, University of Leeds, Leeds LS2 9JT, UK; 3 School of Biomedical Sciences, University of Leeds, Leeds LS2 9JT, UK

**Keywords:** Endothelial, VEGF-A, VEGFR2, UBA1, Ubiquitination, Signal transduction, Angiogenesis

## Abstract

Cell surface receptors can undergo recycling or proteolysis but the cellular decision-making events that sort between these pathways remain poorly defined. Vascular endothelial growth factor A (VEGF-A) and vascular endothelial growth factor receptor 2 (VEGFR2) regulate signal transduction and angiogenesis, but how signaling and proteolysis is regulated is not well understood. Here, we provide evidence that a pathway requiring the E1 ubiquitin-activating enzyme UBA1 controls basal VEGFR2 levels, hence metering plasma membrane receptor availability for the VEGF-A-regulated endothelial cell response. VEGFR2 undergoes VEGF-A-independent constitutive degradation via a UBA1-dependent ubiquitin-linked pathway. Depletion of UBA1 increased VEGFR2 recycling from endosome-to-plasma membrane and decreased proteolysis. Increased membrane receptor availability after UBA1 depletion elevated VEGF-A-stimulated activation of key signaling enzymes such as PLCγ1 and ERK1/2. Although UBA1 depletion caused an overall decrease in endothelial cell proliferation, surviving cells showed greater VEGF-A-stimulated responses such as cell migration and tubulogenesis. Our study now suggests that a ubiquitin-linked pathway regulates the balance between receptor recycling and degradation which in turn impacts on the intensity and duration of VEGF-A-stimulated signal transduction and the endothelial response.

## Abstract (precis)

**Summary:** Ubiquitin-mediated modulation of membrane trafficking and turnover of an endothelial receptor tyrosine kinase (VEGFR2) thus regulates proteolysis, receptor activation, signal transduction and downstream cellular responses. This article has an associated First Person interview with the first author of the paper as part of the supplementary information.

## INTRODUCTION

Vascular endothelial growth factor A (VEGF-A) is an important regulator of animal health and disease (Ferrara, 1999). VEGF-A-stimulated pathological angiogenesis is an important player in chronic inflammatory diseases, cancer and retinopathy (Carmeliet, 2005; Coultas et al., 2005; Ferrara and Kerbel, 2005), whilst insufficient angiogenesis leads to damaged blood vessels, causing tissue ischaemia and heart disease (Ungvari et al., 2010). VEGF binding to a vascular endothelial growth factor receptor (VEGFR) can trigger multiple signal transduction pathways and cellular responses in vascular and non-vascular cells and tissues. In particular, VEGF-A binding to VEGFR2 on endothelial cells causes a diverse range of pro-angiogenic responses (Olsson et al., 2006; Shibuya, 2010). Although highly studied, it is not well understood how the endothelial cell integrates multiple pathways to direct THE sprouting of new blood vessels upon encountering ligands such as VEGF-A.

It is well-established that VEGF-A binding to plasma membrane VEGFR2 causes tyrosine kinase activation and post-translational modifications such as tyrosine trans-autophosphorylation and ubiquitination (Ewan et al., 2006; Koch and Claesson-Welsh, 2012). Ligand-activated VEGFR2 can undergo ubiquitin-linked proteolysis (Bruns et al., 2010; Ewan et al., 2006) which is regulated by E3 ubiquitin ligases such as the proto-oncogene c-Cbl and β-transducin repeat-containing protein (β-TrCP1) (Duval et al., 2003; Shaik et al., 2012; Singh et al., 2007). However, it is unclear how the endothelial cell regulates resting or basal VEGFR2 levels. One possibility is that non-modified, basal VEGFR2 located at the plasma membrane undergoes constitutive endocytosis and delivery to lysosomes for proteolysis. An alternative explanation is that a ubiquitination-dependent mechanism targets basal VEGFR2 for trafficking to degradative compartments such as late endosomes and lysosomes. A recent study has suggested that basal VEGFR2 turnover is regulated by an endosome-associated de-ubiquitinase, USP8 (Smith et al., 2016). Furthermore, the E3 ubiquitin ligase RNF121 controls turnover of newly synthesized VEGFR2 in the secretory pathway (Maghsoudlou et al., 2016). Hence there is an emerging body of evidence that ubiquitination of newly synthesized or basal VEGFR2 trafficking and turnover.

Ubiquitination is a covalent modification involving the formation of an isopeptide bond between the amino terminus of lysine side chains with the free carboxyl terminus of ubiquitin monomers or polymers. The addition of these ubiquitin moieties to a specific protein can alter degradation, intracellular localization and modulate protein activity. Adding such a modification first requires activity of an E1 ubiquitin-activating enzyme, followed by an E2 ubiquitin-conjugating enzyme working in concert with an E3 ubiquitin ligase (Hershko and Ciechanover, 1992). Nine loci within the human genome encode E1-related enzymes which initiate activation and conjugation of a variety of ubiquitin and ubiquitin-like proteins (e.g. SUMO, Nedd8) to target substrates (Pickart, 2001). This study reveals the existence of a novel pathway that programs E1 ubiquitin ligase-dependent modification of basal VEGFR2 to regulate membrane trafficking and proteolysis. Such regulation is important in controlling the endothelial response to VEGF-A by integrating signal transduction, membrane trafficking and cellular responses.

## RESULTS

### UBA1 regulates basal VEGFR2 levels in endothelial cells

**Fig. 1.**
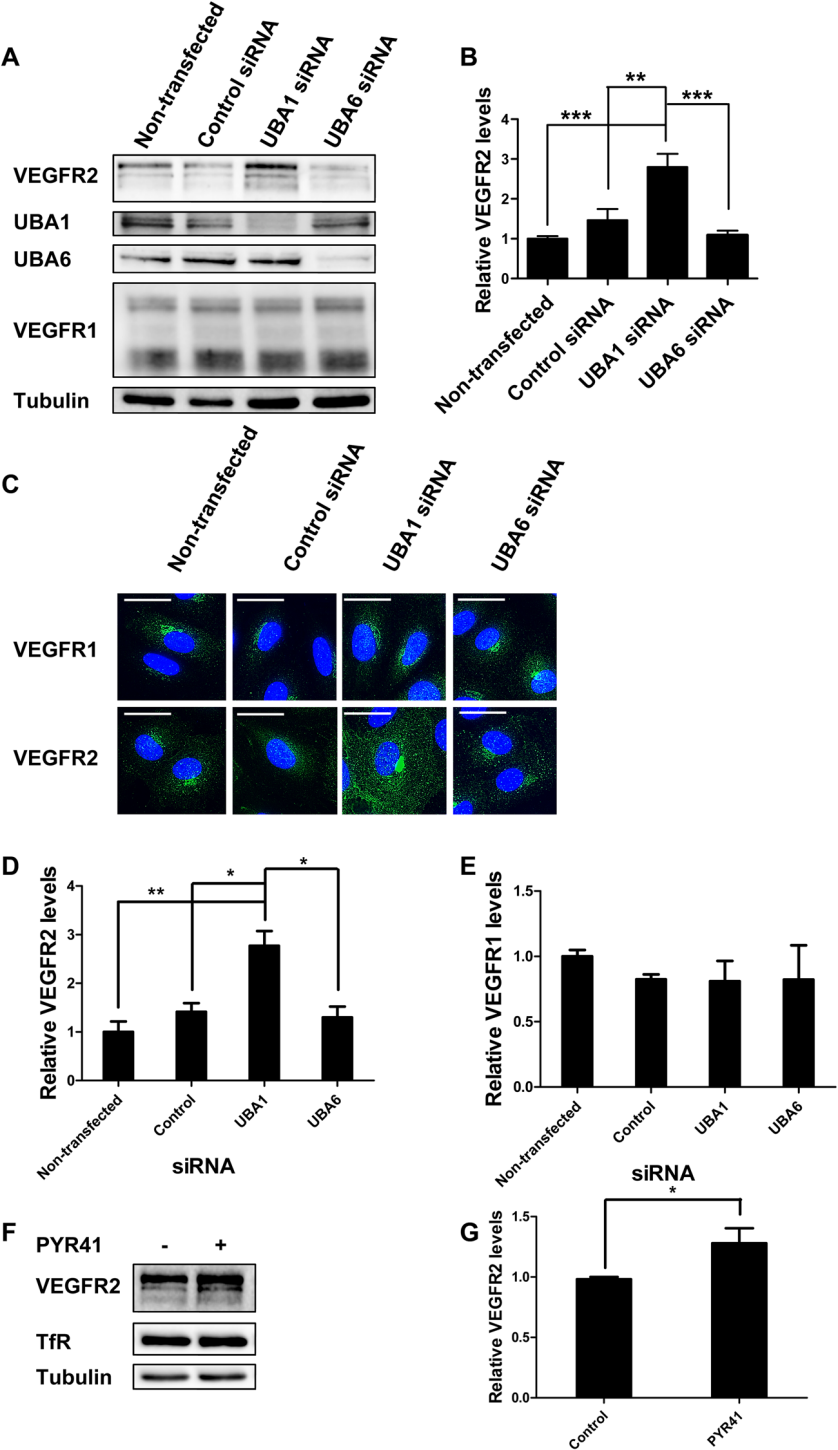
**UBA1 regulates basal VEGFR2 levels.** (A) Endothelial cells treated with non-targeting, UBA1 or UBA6 siRNA were lysed and immunoblotted with antibodies to VEGFR2. (B) Quantification of VEGFR2 levels in non-transfected cells and cells treated with control non-targeting siRNA, UBA1 or UBA6 siRNA. (C) Immunofluorescence analysis on endothelial cells which were either non-transfected, treated with control non-targeting siRNA, UBA1 or UBA6 siRNA, fixed and stained with antibodies to VEGFR1 or VEGFR2 followed by fluorescent species-specific secondary antibodies (green). Nuclei were stained with DNA-binding dye, DAPI (blue). Scale bar: 70 μm. Quantification of (D) VEGFR2 and (E) VEGFR1 levels following immunofluorescence analysis of non-transfected cells, cells treated with control non-targeting siRNA, UBA1 or UBA6 siRNA. (F) Non-treated endothelial cell control lysates (−) compared to treatment (+) with 10 μM PYR41 for 1 h immunoblotted for VEGFR2, transferrin receptor (TfR) and tubulin. (G) Quantification of VEGFR2 levels in endothelial cell control versus PYR41 treatment. In panels B, D, E and G, error bars denote mean±s.e.m. (*n*≥3), with significance denoted as *P<0.05, **P<0.01, ***P<0.001; analysed by one-way ANOVA.

Ligand-stimulated ubiquitination of VEGFR2 facilitates trafficking and degradation in the endosome-lysosome system (Bruns et al., 2010). Previous work has shown that basal VEGFR2 also undergoes proteolysis in primary endothelial cells (Mittar et al., 2009; Ulyatt et al., 2011) but the underlying mechanism was unknown. We hypothesized that ubiquitination of basal VEGFR2 targets this membrane receptor for trafficking and proteolysis. To identify ubiquitin-linked regulators, we evaluated the requirement for E1 ubiquitin-activating enzymes in controlling VEGFR2 levels in human umbilical vein endothelial cells (HUVECs). Experiments revealed that depletion of a major E1 enzyme, UBA1, caused a significant 2.8-fold (*P*<0.01) increase in basal VEGFR2 levels compared to controls (Fig. 1A,B). There was no significant effect on basal levels of VEGFR1, another VEGFR family member (Fig. 1A). Immunofluorescence microscopy analysis showed increased staining for VEGFR2 but not VEGFR1 in UBA1-depleted cells compared to controls (Fig. 1C). Quantification of these staining patterns showed 2.8-fold (*P*<0.05) increase in VEGFR2 levels upon UBA1 depletion (Fig. 1D). Treatment with different UBA1-specific siRNA duplexes consistently increased VEGFR2 levels, as observed using microscopy (Fig. S1A) and quantification of morphological datasets (Fig. S1B). All UBA1-specific siRNAs caused >80% decrease in UBA1 levels (Fig. S1C).

In contrast, VEGFR1 levels were not affected by UBA1 depletion (Fig. 1E). As UBA1 and UBA6 are the only E1 ubiquitin-activating enzymes that regulate ubiquitin attachment to target substrates (Haas et al., 1982; Pelzer et al., 2007), we tested the effects of UBA6 depletion but found that this did not alter VEGFR2 or VEGFR1 levels (Fig. 1A-D). These data suggest that UBA1, but not UBA6, regulates basal VEGFR2 levels.

The pharmacological inhibitor PYR41 irreversibly inhibits E1 enzyme activity whilst showing little or no activity against E2 or E3 enzymes (Yang et al., 2007). Immunoblot analysis of PYR41-treated endothelial cells revealed an increase in basal VEGFR2 levels compared to untreated control (Fig. 1F). Quantification of immunoblot data revealed a ∼30-40% increase in VEGFR2 levels upon PYR41 treatment compared to control (Fig. 1G). Of note, PYR41 effects on VEGFR2 levels were observed within 1 h of treatment, whereas effects of RNAi-mediated depletion of UBA1 were observed 72 h after treatment. Taken together, both RNAi and pharmacological studies suggest a role for UBA1 in regulating basal VEGFR2 levels.

### UBA1 regulates constitutive ubiquitination and degradation of VEGFR2

**Fig. 2.**
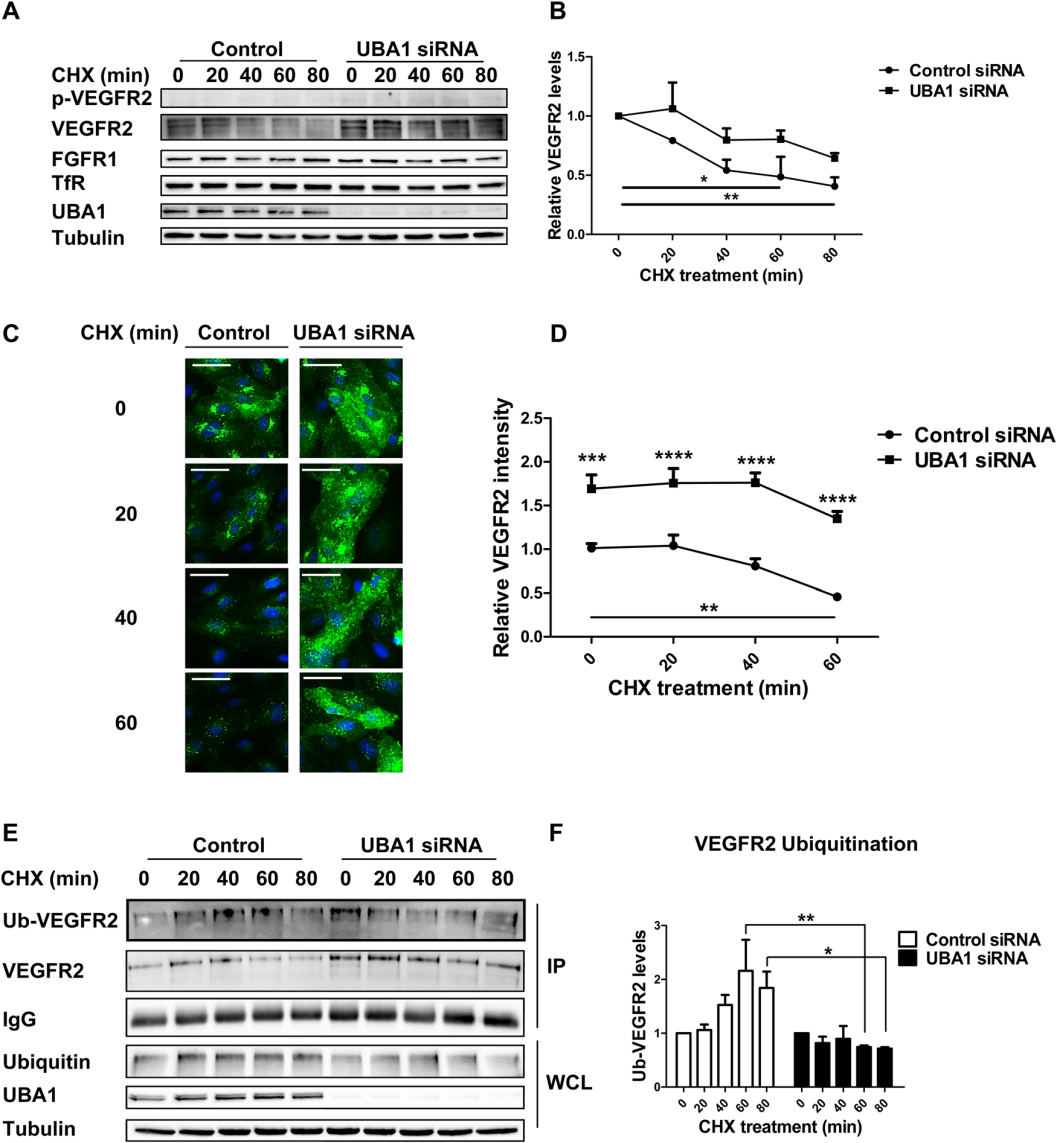
**UBA1 regulates basal VEGFR2 degradation.** (A) HUVECs transfected with non-targeting or UBA1 siRNA were treated with 20 µg/ml cycloheximide (CHX) over a time course of 80 min and immunoblotted using antibodies to phospho-VEGFR2 (pY1175), VEGFR2, FGFR1 and TfR. (B) Quantification of VEGFR2 levels in HUVECs transfected with non-targeting control siRNA or UBA1-specific siRNA combined with 20 µg/ml CHX treatment for 0-80 min presented as decay curves. (C) HUVECs transfected with non-targeting control siRNA or UBA1-specific siRNA were treated with 20 µg/ml CHX over a time course of 0-60 min and visualized using immunofluorescence microscopy by staining with antibodies to VEGFR2 followed by fluorescent species-specific secondary antibodies (green). Nuclei were stained with DNA-binding dye, DAPI (blue). Scale bar: 200 μm. (D) Quantification of VEGFR2 staining from the immunofluorescence microscopy data shown in panel C presented as decay curves. (E) Primary human endothelial cells transfected with non-targeting control siRNA or UBA1-specific siRNA were treated with 20 µg/ml CHX (0-80 min), lysed and VEGFR2 immuno-isolated and probed for ubiquitination status using a ubiquitin-specific antibody. (F) Quantification of ubiquitinated VEGFR2 (Ub-VEGFR2) levels in endothelial cells from the isolation and immunoblotting experiments shown in panel E. Relative Ub-VEGFR2 levels were normalized using total IgG and VEGFR2. IP, immunoprecipitate; WCL, whole cell lysate. In panels B, D, E and F, error bars denote mean±s.e.m. (*n*≥3), with significance denoted as *P<0.05, **P<0.01, ***P<0.001; analysed by two-way ANOVA.

Blocking new protein synthesis using cycloheximide (CHX) enables the monitoring of mature VEGFR2 degradation (Shaik et al., 2012). In these experiments, we combined CHX treatment and RNAi-mediated UBA1 depletion to evaluate UBA1 contribution to VEGFR2 turnover (Fig. 2). Immunoblotting confirmed that basal VEGFR2 levels were elevated upon UBA1 depletion, in the absence of VEGFR2 tyrosine phosphorylation (Fig. 2A). In comparing VEGFR2 turnover to other membrane receptors, UBA1 depletion did not affect basal levels of other cell surface receptors such as fibroblast growth factor receptor 1 (FGFR1) or transferrin receptor (TfR) (Fig. 2A). Quantification of relative protein levels upon CHX treatment revealed that ∼60% of mature VEGFR2 underwent constitutive degradation over an 80 min period (Fig. 2B). In contrast, UBA1-depleted endothelial cells displayed a ∼40% increase in basal VEGFR2 levels prior to CHX addition (t=0 min; Fig. 2B). Upon subjecting UBA1-depleted cells to CHX treatment for different time periods there was a gradual decrease in VEGFR2 levels, however these VEGFR2 levels were still higher (1.6-fold) than in controls with normal UBA1 levels (Fig. 2B). Depletion of UBA1 thus increases steady-state levels of mature VEGFR2 but this is still subject to degradation with similar kinetics to controls (Fig. 2B).

To further assess UBA1 involvement in controlling basal VEGFR2 levels, we analyzed VEGFR2 distribution using immunofluorescence microscopy (Fig. 2C). Quantification of morphological datasets comparing control and UBA1-depleted endothelial cells showed that basal VEGFR2 levels (t=0 min) were ∼60-70% higher in UBA1-depleted cells (Fig. 2D). Under control conditions where new protein synthesis was blocked by CHX, cells displayed a ∼55% decrease in overall VEGFR2 staining after 60 min, compared to the 0-min time point (Fig. 2D). In contrast, CHX-treated and UBA1-depleted cells exhibited only a ∼20% reduction in basal VEGFR2 levels over the same 60 min period compared to the 0-min time point (Fig. 2D). A quantitatively similar effect of UBA1 depletion on mature VEGFR2 was also seen in a second vascular cell type, human dermal microvascular endothelial cells (HDMECs) (Fig. S2A). Basal levels of mature VEGFR2 in HDMECs were elevated by ∼20-30% after UBA1 depletion and did not decrease significantly upon CHX treatment for up to 80 min in comparison to controls (Fig. S2B). These data show UBA1 regulates basal VEGFR2 levels in different endothelial cells derived from veins (HUVECs) and capillaries (HDMECs).

One likely explanation for UBA1-mediated regulation is that basal VEGFR2 undergoes ubiquitination by a novel pathway. To test this idea, mature VEGFR2 from control or UBA1-depleted endothelial cells was immunoprecipitated and ubiquitination status evaluated over a 0-80 min time course of CHX treatment (Fig. 2E). At the 0-min time point, relative ubiquitination compared to total VEGFR2 levels was not significantly different in UBA1-depleted cells compared to controls (Fig. 2F). However, during the time course of CHX treatment it was noticeable that ubiquitinated VEGFR2 levels were significantly higher in control cells than in UBA1-depleted cells (Fig. 2E). After 40 min of CHX treatment, control cells exhibited 2.9-fold (*P*<0.01) greater levels of ubiquitinated VEGFR2 compared to UBA1-depleted cells (Fig. 2F). Thus reduction in UBA1 levels decreased basal VEGFR2 ubiquitination.

### UBA1 regulates basal VEGFR2 recycling

**Fig. 3.**
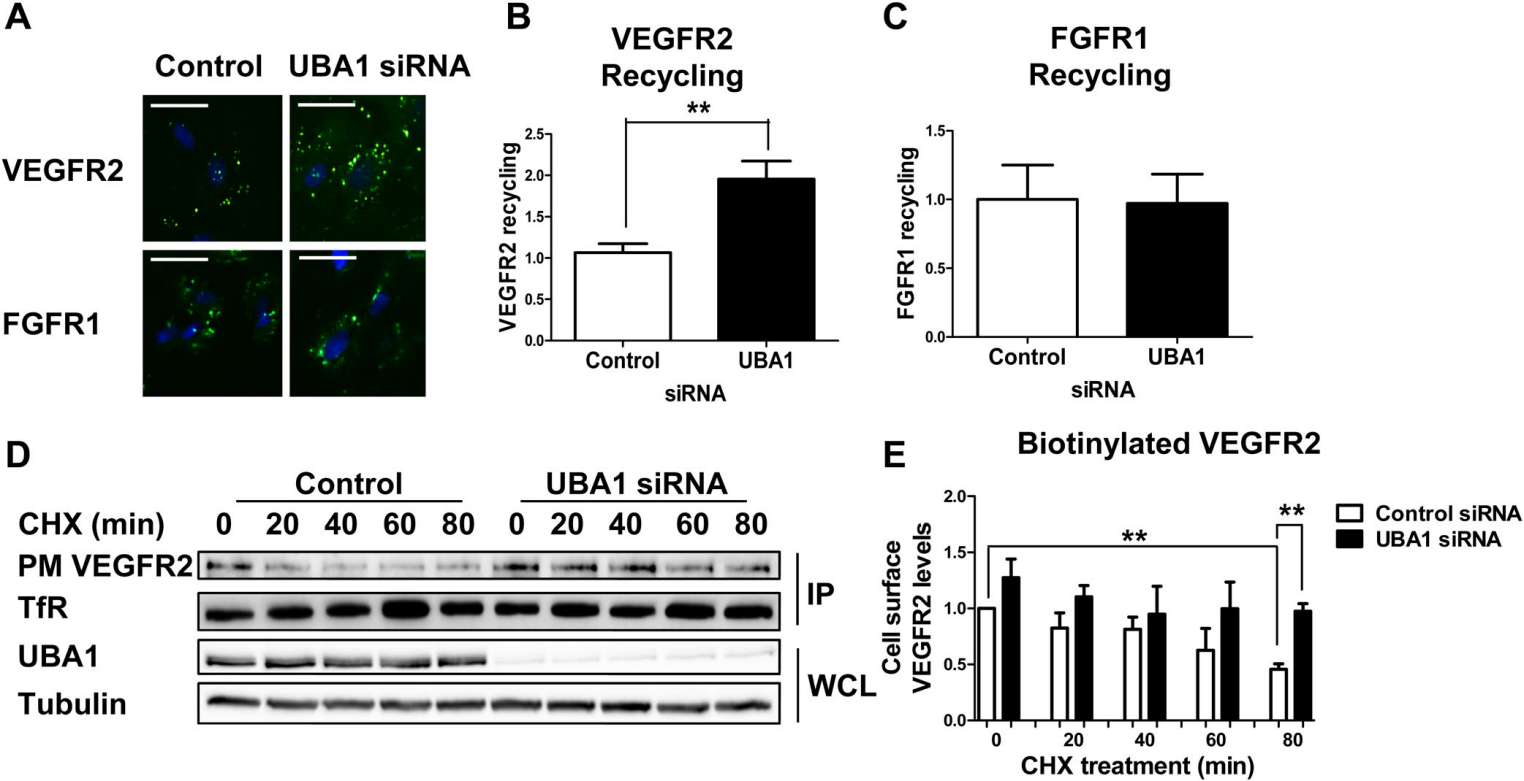
**UBA1 regulates basal ubiquitination, recycling and plasma membrane levels of VEGFR2.** (A) Endothelial cells transfected with non-targeting control siRNA or UBA1-specific siRNA were incubated with antibodies to the extracellular domains of VEGFR2 or FGFR1 for 30 min at 37°C prior to acid-wash to strip cell surface antibodies, and incubation with fluorescent species-specific secondary antibodies for 30 min at 37°C (green). Cells were fixed prior to staining with DAPI (blue). Only VEGFR2 or FGFR1 that underwent plasma membrane-to-endosome-to-plasma membrane recycling is visible. Scale bar: 200 μm. Quantification of (B) VEGFR2 and (C) FGFR1 recycling in endothelial cells transfected with non-targeting control siRNA or UBA1-specific siRNA. (D) Endothelial cells transfected with non-targeting control siRNA or UBA1-specific siRNA were treated with 20 µg/ml CHX for 0-80 min before cell surface proteins were biotinylated, isolated and immunoblotted for plasma membrane VEGFR2 (PM VEGFR2), transferrin receptor (TfR), UBA1 and tubulin. IP, immunoprecipitate; WCL, whole cell lysate. (E) Quantification of immunoblot data shown in panel D with relative values for cell surface VEGFR2 levels denoted in the histogram. In panels B and E, error bars denote mean±s.e.m. (*n*≥3), with significance denoted as *P<0.05, **P<0.01; analysed using one-way ANOVA (panel B) and two-way ANOVA (panel E).

Ubiquitination at the plasma membrane frequently precedes receptor tyrosine kinase (RTK) endocytosis, delivery to early endosomes and further trafficking to lysosomes for terminal degradation (Clague and Urbé, 2001; Ewan et al., 2006; Haglund and Dikic, 2012). However, RTK de-ubiquitination in early or late endosomes could also enable recycling from endosome-to-plasma membrane (Clague and Urbé, 2006). Such features have previously been observed in this system with VEGF-A-stimulated VEGFR2 ubiquitination promoting trafficking to late endosomes, linked to terminal degradation in lysosomes (Bruns et al., 2010; Ewan et al., 2006). Furthermore, VEGFR2 can also undergo substantial constitutive ligand-independent recycling via endosomes (Jopling et al., 2011). Another RTK such as FGFR1 undergoes similar constitutive recycling (Hausott et al., 2012). One possibility is that upon UBA1 depletion, VEGFR2 undergoes decreased basal ubiquitination that in turn permits increased endosome-to-plasma membrane recycling. To test this idea, we used a VEGFR2 recycling assay (Jopling et al., 2011) in which control and UBA1-depleted endothelial cells were incubated with antibodies specific for the extracellular domains of VEGFR2 or FGFR1. Constitutive RTK endocytosis and recycling was then monitored using accessibility of VEGFR2-antibody and FGFR1-antibody complexes to a pulse of labeled secondary antibody. Only VEGFR2-antibody or FGFR1-antibody complexes that underwent endocytosis followed by endosome-to-plasma membrane recycling were detected in this assay (Fig. 3A). Compared to control cells, UBA1-depleted endothelial cells displayed a twofold (*P*<0.01) increase in endosome-to-plasma membrane recycling of non-activated VEGFR2 (Fig. 3B). In contrast, FGFR1 recycling was not significantly affected by UBA1 depletion (Fig. 3C).

One possibility is that increased endosome-to-plasma membrane recycling after UBA1 depletion caused an overall net increase in plasma membrane VEGFR2 levels. To test this idea, new protein synthesis was blocked with CHX and a cell surface biotinylation assay was performed to monitor the plasma membrane pool. Immunoblot analysis showed that basal VEGFR2 plasma membrane levels in UBA1-depleted cells were ∼25% higher than in control cells (t=0 min, Fig. 3D,E). Another cell surface receptor, transferrin receptor, was not significantly affected (Fig. 3D). In control cells treated with CHX, there was a ∼55% decrease in levels of plasma membrane VEGFR2 (Fig. 3E). In contrast, under the same CHX treatment of UBA1-depleted cells there was a less marked (∼24%) decrease in plasma membrane VEGFR2 levels (Fig. 3E). These data suggest that loss of UBA1 causes an increase in plasma membrane VEGFR2 levels and is also consistent with increased VEGFR2 recycling from endosome-to-plasma membrane.

### UBA1 regulates VEGFR2 trafficking to endosomes and lysosomes

**Fig. 4.**
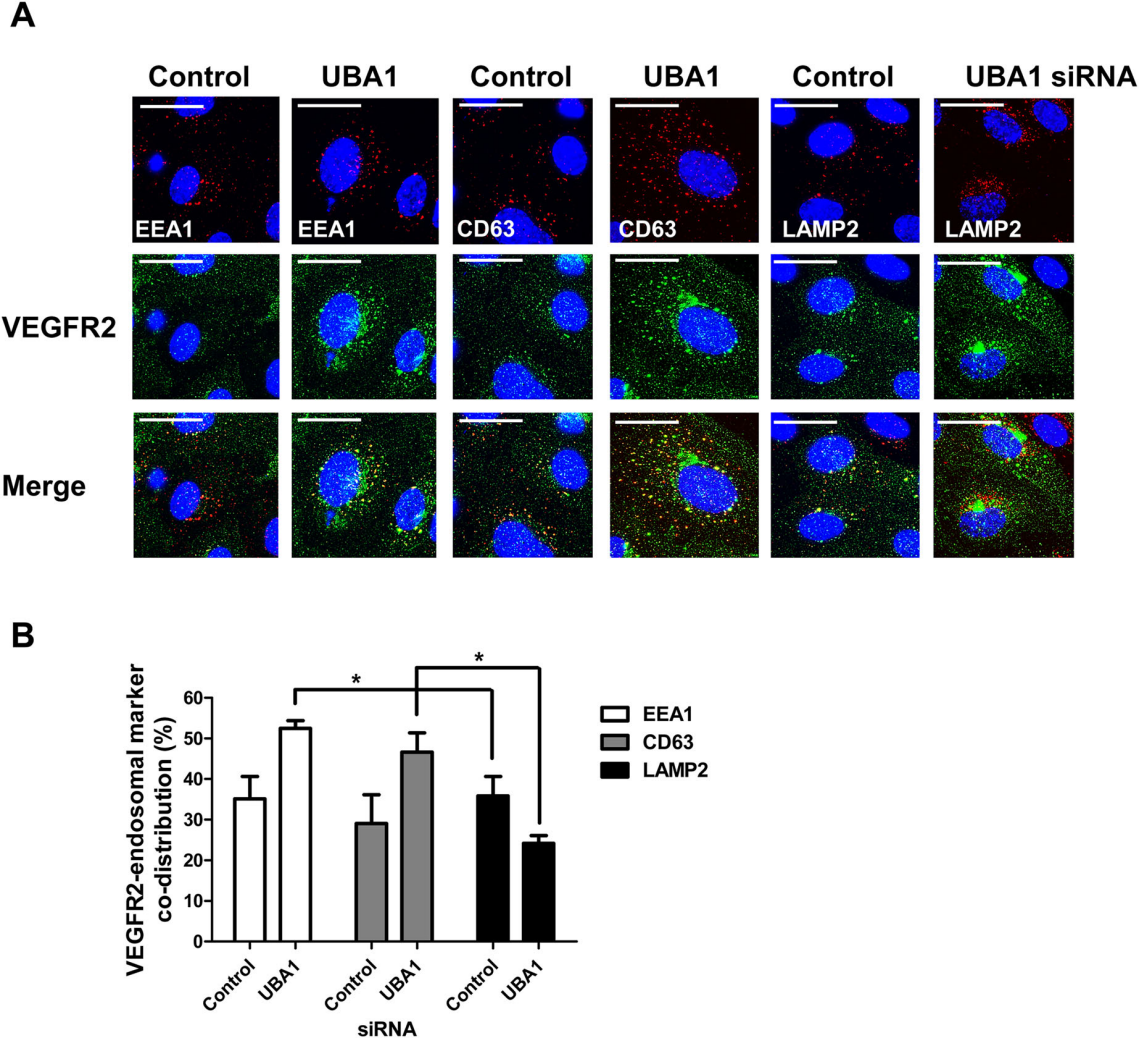
**UBA1 depletion perturbs VEGFR2 endosome-lysosome dynamics.** (A) Endothelial cells transfected with non-targeting or UBA1 siRNA were processed for immunofluorescence microscopy using antibodies to VEGFR2 (green), EEA1 (red), CD63 (red) or LAMP2 (red) followed by fluorescent species-specific secondary antibodies. Nuclei were stained with DNA-binding dye, DAPI (blue). Scale bar: 70 μm. (B) Quantification of VEGFR2 co-distribution with early endosome (EEA1), late endosome (CD63) and lysosome (LAMP2) markers in endothelial cells transfected with non-targeting control siRNA or UBA1-specific siRNA. Error bars denote mean±s.e.m. (*n*≥3), with significance denoted as *P<0.05; analysed using two-way ANOVA.

VEGFR2 undergoes endocytosis, delivery to endosomes and recycling back to the plasma membrane or commitment for terminal degradation in late endosomes and lysosomes (Ewan et al., 2006; Jopling et al., 2014). If UBA1 depletion affects VEGFR2 membrane dynamics, loss of UBA1 would be expected to alter VEGFR2 distribution within these compartments. To ascertain this, we compared VEGFR2 co-distribution with EEA1, CD63 or LAMP2 (Fig. 4). UBA1-depleted endothelial cells exhibited a ∼50% increase in VEGFR2 co-distribution with the early endosome marker EEA1 compared to controls (Fig. 4A,B). There was a similar increase in VEGFR2 co-distribution with the late endosome marker CD63 (Fig. 4B). In contrast, UBA1-depleted cells exhibited a ∼34% decrease in VEGFR2 co-distribution with the lysosome marker LAMP2 compared to control (Fig. 4B). These data suggest that decreased UBA1 levels alter VEGFR2 distribution within the endosome-lysosome network.

### UBA1 regulates VEGF-A-stimulated signal transduction

**Fig. 5.**
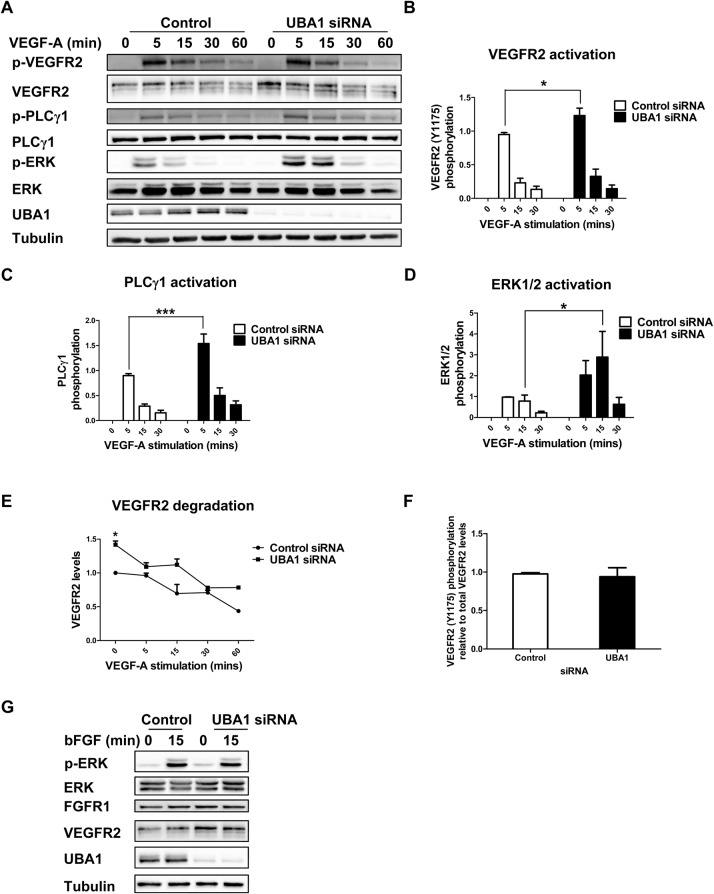
**UBA1 depletion causes an increase in VEGF-A-stimulated signal transduction.** (A) Endothelial cells transfected with non-targeting control siRNA or UBA1-specific siRNA were stimulated with 25 ng/ml VEGF-A for specific time periods (0-60 min), lysed and immunoblotted for phospho-VEGFR2 (Y1175), phospho-PLCγ1 (Y783), phospho-ERK1/2 (T202/Y204), VEGFR2, ERK1/2, UBA1 and tubulin. Quantification of (B) phospho-VEGFR2, (C) phospho-PLCγ1, (D) phospho-ERK1/2, and (E) VEGFR2 levels in endothelial cells transfected with non-targeting control siRNA or UBA1-specific siRNA and treated with 25 ng/ml VEGF-A for the time periods indicated. Relative levels for each signal were normalized against tubulin. (F) Quantification of phospho-VEGFR2 (Y1175) levels after 5 min VEGF-A stimulation and normalized against total VEGFR2 levels in control or UBA1-depleted endothelial cells. (G) Endothelial cells transfected with non-targeting control siRNA or UBA1-specific siRNA were treated with 25 ng/ml basic FGF (bFGF), lysed and immunoblotted for phospho-ERK1/2 (T202/Y204), VEGFR2, FGFR1, UBA1 and tubulin. In panels B-F, error bars denote mean±s.e.m. (*n*≥3), with significance denoted as *P<0.05, ***P<0.001; analysed using two-way ANOVA.

VEGF-A binding to plasma membrane VEGFR2 stimulates multiple signal transduction pathways (Koch et al., 2011; Zhang et al., 2008). Our experiments now show that UBA1 depletion leads to a net increase in plasma membrane VEGFR2; this could modulate VEGF-A-stimulated signal transduction. To test this idea, control and UBA1-depleted endothelial cells were stimulated with VEGF-A before probing downstream signaling events using quantitative immunoblotting (Fig. 5). VEGFR2 activation is exemplified by phosphorylation on cytoplasmic residue Y1175 (Koch et al., 2011); this was clearly evident in both control and UBA1-depleted cells (Fig. 5A). However, UBA1 depletion caused a significant ∼30% increase in VEGFR2-pY1175 levels (Fig. 5B). Plasma membrane VEGFR2 activation is also linked to recruitment of phospholipase Cγ1 (PLCγ1) followed by tyrosine phosphorylation on residue Y783 and increased phospholipase activity (Koch et al., 2011). UBA1-depleted cells exhibited enhanced PLCγ1 phosphorylation (Fig. 5A) with ∼43% increase in PLCγ1-pY783 levels (Fig. 5C). A key feature of VEGF-A-stimulated signaling is activation of the canonical mitogen-activated protein kinase (MAPK) pathway leading to phosphorylation and activation of extracellular signal-regulated kinase enzymes 1 and 2 (ERK1/2) (Koch and Claesson-Welsh, 2012). VEGF-A stimulation caused a 3.7-fold (*P*<0.05) increase in ERK1/2 phosphorylation in UBA1-depleted endothelial cells compared to controls (Fig. 5A,D). UBA1-depleted cells contained ∼40% higher basal VEGFR2 levels. Surprisingly, the kinetics of VEGF-A-stimulated VEGFR2 degradation were not significantly affected by UBA1 depletion (Fig. 5A,E). Thus, UBA1 is not required for VEGF-A-stimulated VEGFR2 degradation.

These data suggest that increased VEGFR2 phosphorylation in UBA1-depleted cells (Fig. 5B) was due to an overall net increase in plasma membrane VEGFR2 levels rather than effects on VEGFR2 activation at the individual receptor level (Fig. 5F). To test whether UBA1 depletion affects other RTK signal transduction events, UBA1-depleted endothelial cells were subjected to a time-course of bFGF which is known to activate FGFR1-regulated MAPK signal transduction in endothelial cells. Both control and UBA1-depleted cells exhibited similar responses to bFGF stimulation such as ERK1/2 activation (Fig. 5G). Thus UBA1 regulates signal transduction by VEGFR2 but not FGFR1.

### Basal VEGFR2 turnover regulates VEGF-A-dependent endothelial cell tubulogenesis

**Fig. 6.**
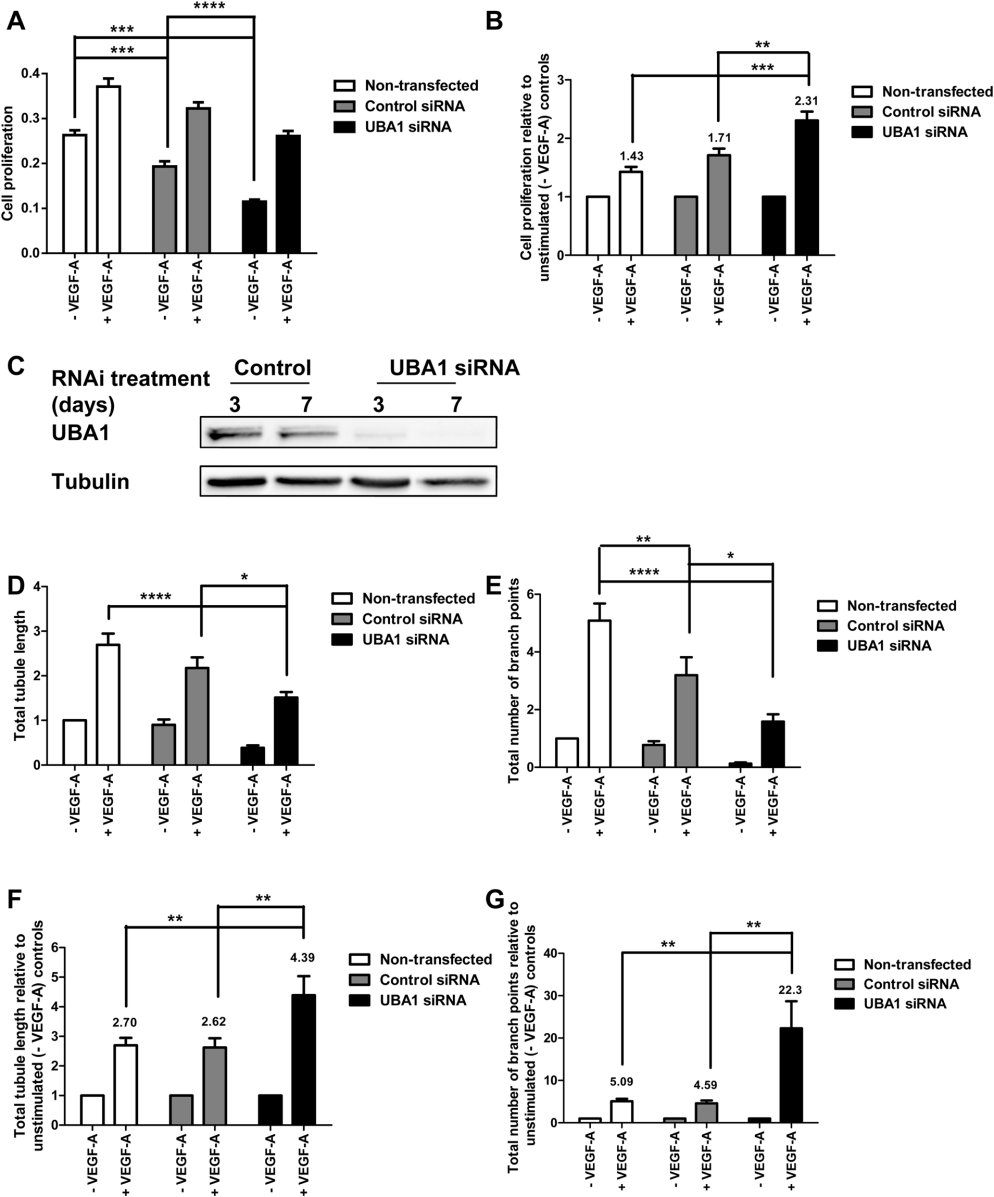
**UBA1 influences VEGF-A-stimulated endothelial cell proliferation and tubulogenesis.** (A) Endothelial cells transfected with non-targeting control siRNA or UBA1-specific siRNA were analyzed for cell proliferation using a bromodeoxyuridine (BrdU) incorporation assay. (B) Quantification of VEGF-A-stimulated cell proliferation, expressed as fold increase over the corresponding values for non-stimulated cells. (C) Endothelial cells transfected with non-targeting control siRNA or UBA1-specific siRNA for 3 or 7 days were lysed and UBA1 levels assessed by immunoblotting. Quantification of VEGF-A-stimulated endothelial tubulogenesis (see Materials and Methods) by evaluating (D) total tubule length and (E) total number of branch points relative to the non-transfected, non-stimulated (-VEGF-A) condition. Quantification of VEGF-A-stimulated endothelial tubulogenesis by evaluating (F) total tubule length, and (G) total number of branch points expressed as fold increase over corresponding values for non-stimulated cells in non-transfected, control siRNA-treated or UBA1-treated cells. In panels A, B and D-G, error bars denote mean±s.e.m. (*n*≥3), with significance denoted as *P<0.05, **P<0.01, ***P<0.001, ****P<0.0001; analysed using two-way ANOVA.

UBA1 is the principal E1 enzyme in human cells and is likely to be involved in many cellular processes (Groen and Gillingwater, 2015). Depletion of UBA1 caused 2.4-fold (*P*<0.001) decrease in endothelial cell proliferation in the absence of VEGF-A (Fig. 6A). However, remaining viable endothelial cells showed a 2.3-fold increase in VEGF-A-stimulated proliferation compared to a 1.7-fold increase in control cells (*P*<0.01) (Fig. 6B). Signal transduction by VEGF-A-activated VEGFR2 promotes new vascular tube formation by endothelial cells, an essential feature in angiogenesis (Ferrara, 1999). Immunoblotting confirmed that UBA1-specific siRNA transfection was effective at depleting endothelial UBA1 levels for extended periods (Fig. 6C), corresponding to the 7-day duration of the tubulogenesis assay. UBA1-depleted cells exhibited lower tubule length (Fig. 6D) and number of tubule branch points (Fig. 6E) in absolute numbers compared to non-transfected or control siRNA-transfected controls. However, VEGF-A stimulation of UBA1-depleted cells increased tubule length 4.4-fold (Fig. 6F) and branch point number 22.3-fold when compared to UBA1-depleted cells in the absence of VEGF-A (Fig. 6F). These effects were substantially higher than the VEGF-A-stimulated 2.6-fold (*P*<0.01) increase in tubule length (Fig. 6F) and 4.6-fold (*P*<0.001) increase in branch point number (Fig. 6G) for control siRNA-transfected controls. These findings show that UBA1 has functional impact on VEGF-A-stimulated endothelial tubulogenesis.

### VEGF-A-stimulated endothelial cell migration is elevated by decreased UBA1 levels

**Fig. 7.**
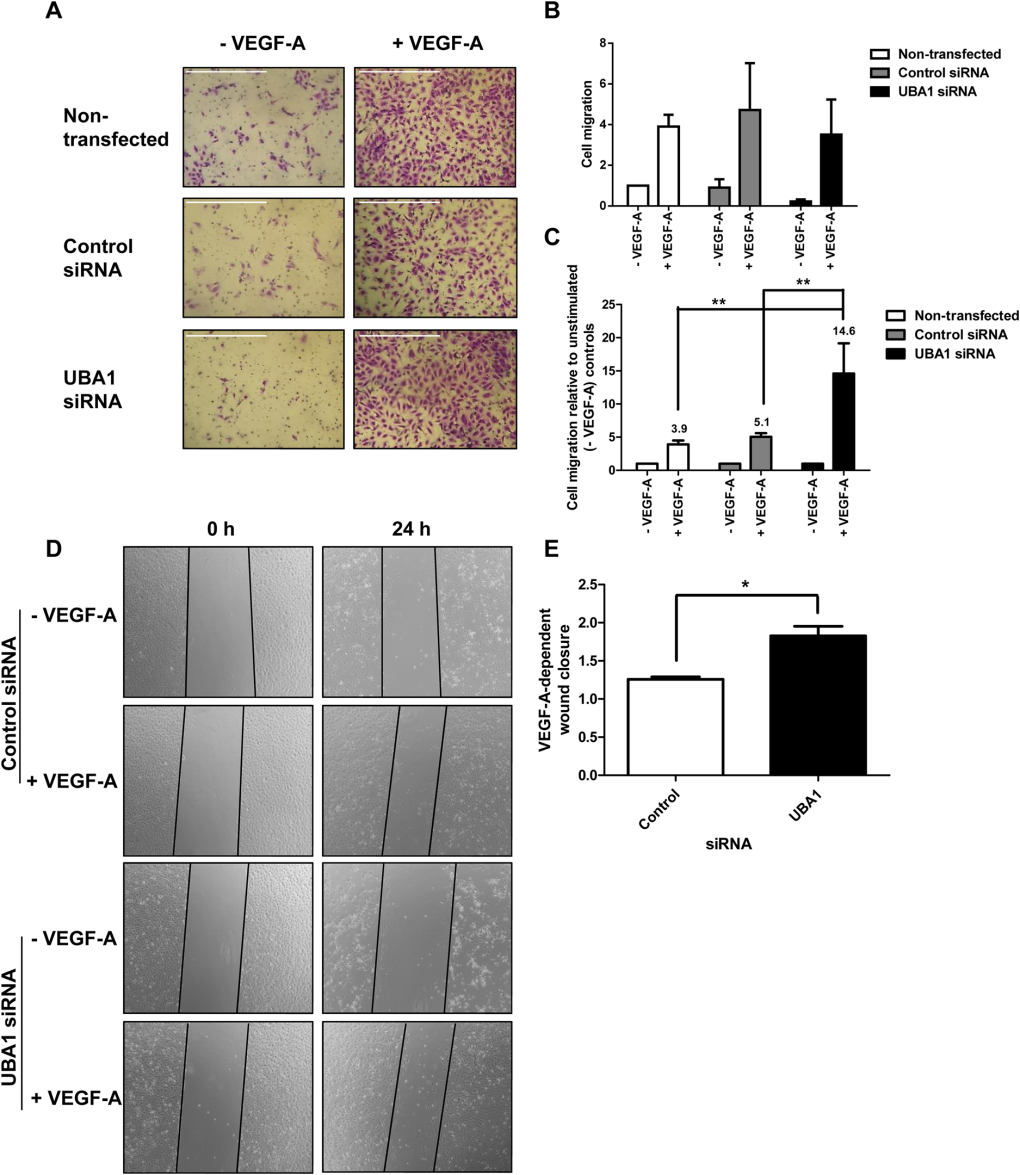
**UBA1 influence on VEGF-A-stimulated endothelial cell migration and monolayer wound closure.** (A) Non-transfected endothelial cells or cells transfected with non-targeting control siRNA or UBA1-specific siRNA were seeded into Transwell filters and stimulated with VEGF-A (25 ng/ml) for 24 h, then fixed and stained. Scale bar: 1000 μm. (B) Quantification of endothelial cell migration relative to the non-transfected, non-stimulated (-VEGF-A) condition. (C) Quantification of the VEGF-A-dependent increase in cell migration expressed as fold increase over the corresponding values for non-stimulated cells. (D) Endothelial cell monolayers transfected with non-targeting control siRNA or UBA1-specific siRNA were wounded (0 h), treated with 25 ng/ml VEGF-A for 24 h and images recorded by microscopy. (E) Quantification of VEGF-A-stimulated endothelial monolayer wound closure in cells transfected with non-targeting control siRNA or UBA1-specific siRNA. In panels B, C and E, error bars denote mean±s.e.m. (*n*≥3), with significance denoted as *P<0.05, **P<0.01; analysed using two-way ANOVA.

VEGF-A-dependent signaling also stimulates endothelial cell migration (Fearnley et al., 2014; Smith et al., 2015a,b). To test the role of UBA1, we analyzed control and UBA1-depleted endothelial cells for migration towards VEGF-A (Fig. 7A). Quantification of these images showed that UBA1 depletion caused an overall decrease in non-stimulated and VEGF-A-stimulated endothelial cell migration compared to non-transfected or control siRNA-transfected cells (Fig. 7B). However, comparison of non-stimulated versus VEGF-A-stimulated migration of UBA1-depleted cells showed 14.6-fold increase in VEGF-A-stimulated endothelial cell migration (Fig. 7C). This effect was 3.7-fold (*P*<0.001) higher than the VEGF-A-stimulated migration exhibited by control siRNA-transfected cells (Fig. 7C). We also tested the VEGF-A-stimulated closure of a wounded endothelial cell monolayer which represents both cell proliferation and migration. There was a significant VEGF-A-stimulated re-occupation of the wounded area by UBA1-depleted cells in comparison to non-transfected and control siRNA-treated cells (Fig. 7D). This experiment showed a ∼31% increase in VEGF-A-stimulated wound closure in UBA1-depleted cells compared to control siRNA-treated cells (Fig. 7E). These data show that loss of UBA1 elevates the endothelial cell response to VEGF-A which is reflected reflected by endothelial endothelial cell migration and monolayer wound closure.

## DISCUSSION

Our study now provides compelling evidence for a ubiquitin-linked pathway which regulates basal VEGFR2 levels and impacts on VEGF-A-stimulated signal transduction and multiple cellular responses. Our findings support the existence of a mechanism whereby cells adjust the net pool of plasma membrane VEGFR2, thus controlling RTK-mediated signal transduction and the cellular response to extracellular ligands such as VEGF-A. This ligand-independent regulatory pathway mediates VEGFR2 availability at the plasma membrane for VEGF-A-stimulated signal transduction. The E1 enzyme, UBA1, regulates basal plasma membrane VEGFR2 levels which influence VEGF-A-stimulated activation of PLCγ1 and ERK1/2 signal transduction pathways. A key point is that UBA1 influences the pool of plasma membrane VEGFR2 which in turn dictates net VEGFR2 activation.

VEGFR2 ubiquitination plays key roles in membrane trafficking and degradation but previous work has focused on VEGF-stimulated responses (Bruns et al., 2010; Ewan et al., 2006; Smith et al., 2015a,b). Our study now highlights a mechanism involving UBA1 which controls basal VEGFR2 levels and VEGF-A-stimulated cellular responses. This type of RTK ubiquitination is closely linked to trafficking as highlighted by perturbation of VEGFR2 endosome-to-plasma membrane recycling when UBA1 levels are depleted. Under these conditions, VEGFR2 showed increased co-distribution with endosomes but reduced co-distribution with lysosomes. Trafficking of other plasma membrane receptors such as transferrin receptor and another RTK (FGFR1) did not show UBA1-dependence, suggesting this UBA1-regulated pathway has specificity for a subset of proteins which includes VEGFR2. Nonetheless, such ubiquitin-linked regulation of basal VEGFR2 has important consequences for VEGF-A-stimulated cellular responses such as endothelial tubulogenesis, migration and proliferation: there is clear elevation in VEGF-A-stimulated pro-angiogenic responses upon UBA1 depletion.

Ligand-stimulated ubiquitination of VEGFR2 programs terminal degradation in lysosomes (Ewan et al., 2006). Conflicting studies implicate E3 ligases Cbl proto-oncogene E3 ubiquitin protein ligase (c-Cbl) and β-transducin repeat containing E3 ubiquitin protein ligase (β-TrCP1) in VEGF-A-stimulated proteolysis of VEGFR2 (Bruns et al., 2010; Duval et al., 2003; Murdaca et al., 2004; Shaik et al., 2012; Singh et al., 2007). Furthermore, differences in VEGFR1 and VEGFR2 proteolysis under either resting (Mittar et al., 2009) or hypoxic (Ulyatt et al., 2011) conditions suggest that endothelial cells exploit VEGFR availability to fine-tune the cellular response to VEGF-A. Recent studies have also highlighted ligand-independent VEGFR2 de-ubiquitination linked to the de-ubiquitinase USP8 that controls membrane trafficking, recycling and proteolysis (Smith et al., 2015a,b). Interestingly, kinase-independent regulation of RTK function is highlighted by the discovery that constitutive binding of cytosolic adaptors such as growth factor receptor-binding protein 2 (Grb2) to basal FGFR2 regulates ligand-independent activation of downstream signaling pathways (Lin et al., 2012). In addition, ligand-independent ubiquitination and endocytosis of EGFR involves the Hrs endocytic adaptor protein (Katz et al., 2002) that is found on a subset of early endosomes. There is also a new kinase-independent autophagic role for EGFR (Tan et al., 2015). These diverse studies emphasize how ligand-independent control of RTK turnover and function can impact on ligand-stimulated cellular responses.

UBA1 is an essential cellular enzyme expressed by many cells and tissues and is functionally implicated in multiple pathways including DNA replication. Notably, suppression of UBA1 activity in Schwann cells is linked to spinal muscular atrophy (Aghamaleky Sarvestany et al., 2014; Sugaya et al., 2015). Other studies have identified UBA1 as a novel target for the treatment of hematological malignancies (Xu et al., 2010; Yang et al., 2007). UBA1-mediated surveillance of disease-linked responses could thus be utilized for controlling RTK levels and cellular responses in different tissues. The potential for UBA1 in cell proliferation and disease is highlighted in the profiling of certain cancers (e.g. prostate cancer) which show reduced UBA1 expression (www.proteinatlas.org). One mechanism employed by cancerous cells could be down-regulation of UBA1 expression to stimulate tumor angiogenesis. By providing a UBA1-regulated mechanism to control basal VEGFR2 availability which impacts on signal transduction and cellular responses, our study provides a non-canonical pathway that is unique to the established model for ligand-stimulated RTK ubiquitination, trafficking and proteolysis. Our findings provide a new understanding of ubiquitin-linked regulation of VEGF-regulated outcomes and could be of use to new strategies that target angiogenesis in diverse disease states.

## MATERIALS AND METHODS

### Cell culture and materials

Primary HUVECs were cultured as previously described (Fearnley et al., 2014; Howell et al., 2004), HDMECs and appropriate growth media were from PromoCell (Heidelberg, Germany). Purified primary and secondary antibodies were typically used at 1 µg/ml for microscopy and at 0.1 µg/ml for immunoblotting. These antibodies were goat anti-VEGFR2 (R&D Systems, Minneapolis, USA), rabbit anti-phospho-VEGFR2 (Y1175), rabbit anti-UBA1 (Cell Signaling Technologies, Danvers, USA), rabbit antibodies to native and phosphorylated PLCγ1 (Y783), rabbit anti-ERK1/2, mouse anti-phospho-ERK1/2 (T202, Y204), mouse anti-α-tubulin (Santa Cruz Biotechnology, USA), mouse anti-transferrin receptor (TfR), mouse FK2 anti-ubiquitin (Affiniti Research Products, Exeter, UK), mouse anti-EEA1 (BD Biosciences, California, USA), mouse anti-CD63 (Abcam, Cambridge, UK), mouse anti-LAMP2 (Santa Cruz, USA), HRP-conjugated secondary antibodies (Thermo Fisher, Loughborough, UK) and Alexa Fluor-conjugated secondary antibodies (ThermoFisher). Endothelial cell growth medium (PromoCell), non-targeting and UBA1 siRNA duplexes (GE Dharmacon, UK) and recombinant human VEGF-A_165_ (Genentech Inc., San Francisco, USA) were obtained as stated. Chemicals were obtained from Sigma-Aldrich (Poole, UK) or Thermo Fisher (Loughborough, UK).

### Immunoblotting and immunofluorescence microscopy

Endothelial cells were serum starved in MCDB131 (Thermo Fisher) for 2 h prior to treatment with 25 ng/ml VEGF-A_165_ (0-60 min), 20 μg/ml CHX (0-80 min) or 10 μM PYR41 (1 h) and lysed for immunoblotting or immunoprecipitation studies. Cells were lysed in 2% (w/v) SDS and run on a 10% SDS-PAGE gel at 120 V for 90 min. Proteins were transferred onto nitrocellulose membrane at 300 mA for 3 h and incubated in primary antibodies overnight prior to incubation in HRP-conjugated secondary antibodies for 1 h and detection using enhanced chemiluminescence. Immunoblots were quantified, normalized against tubulin and made relative to the control (i.e. the control siRNA 0 min condition) for representation on graphs. For immunofluorescence analysis, HUVECs were seeded in 96-well plates or on cover-slips before fixation, permeabilization in 0.1% (w/v) Triton X-100, incubation with primary antibodies and visualization by incubation in Alexa Fluor488- or 594-conjugated secondary antibodies and DAPI. Images were acquired using an EVOS-fl inverted digital microscope (Thermo Fisher) at 20× magnification or a wide-field deconvolution microscope DeltaVision (Applied Precision Inc., Issaquah, USA) at 60× magnification at room temperature. Fluorescence intensity (integrated density) and co-localization (co-localization threshold plugin) were quantified using NIH ImageJ (https://imagej.nih.gov/ij/download.html).

### Immunoprecipitation analysis

HUVECs were serum starved for 2 h prior to CHX treatment or VEGF-A stimulation, lysed in buffer [150 mM NaCl, 50 mM Tris-HCl, pH 7.4, 0.1% (w/v) SDS, 0.5% (w/v) sodium deoxycholate, 2 mM EDTA, 1% (v/v) NP-40, 50 mM NaF, 1 mM PMSF, 10 mM iodoacetamide], incubated with 1 μg/ml goat anti-VEGFR2 for 2 h and immuno-isolated with protein G-agarose beads before SDS-PAGE and immunoblot analysis.

### Plasma membrane protein recycling assay

HUVECs were incubated in primary antibody to VEGFR2 or FGFR1 for 30 min at 37°C. Cell surface-bound primary antibody was stripped by washing in acidic MCDB131 medium (pH 2.0) at 4°C. Cells were incubated in secondary antibody (anti-sheep Alexa Fluor488) for 30 min at 37°C and fixed for 5 min at 37°C before incubation with 1 μg/ml DAPI to visualize nuclear DNA. Only cell surface VEGFR2 that had bound primary antibody and undergone internalization and subsequent recycling would be available to bind secondary antibody after acid-washing. Thus, only VEGFR2 that recycled one or more times was visualized. Images were acquired using an EVOS-fl inverted digital microscope at 20× magnification at room temperature. Fluorescence intensity was quantified using NIH ImageJ (https://imagej.nih.gov/ij/download.html).

### Cell surface biotinylation

HUVECs were serum starved for 2 h prior to CHX treatment, washed in ice-cold PBS, cell surface biotinylated by incubation with 0.25 mg/ml biotin in buffer (2 mM CaCl_2_, 2 mM MgCl_2_, PBS) for 45 min, washed in TBS to quench biotinylation and lysed in NP-40 lysis buffer [1% (v/v) NP-40, 50 mM Tris-HCl, pH 7.5, 150 mM NaCl, 1 mM PMSF]. Cell surface proteins were isolated using NeutraAvidin agarose beads before SDS-PAGE and immunoblotting.

### Protein depletion using RNAi

Endothelial cells were reverse transfected in 6- or 96-well plates with 4 pooled siRNA duplexes (SMARTpool siRNA, GE Dharmacon) as follows. 20 nM non-targeting control siRNA: 5′-UGGUUUACAUGUCGACUAA-3′; 5′-UGGUUUACAUGUUGUGUGA-3′; 5′-UGGUUUACAUGUUUUCUGA-3′; 5′-UGGUUUACAUGUUUUCCUA-3′. 20 nM UBA1 siRNA: 5′-GCGUGGAGAUCGCUAAGAA-3′; 5′-CCUUAUACCUUUAGCAUCU-3′; 5′-CCACAUAUCCGGGUGACAA-3′; 5′-GAAGUCAAAUCUGAAUCGA-3′.

All siRNA duplexes were used according to the manufacturer's instructions (GE Dharmacon). Endothelial cells were incubated for 6 h with siRNA duplexes using a previously described lipid-based transfection protocol (Fearnley et al., 2014). After 72 h, cells were processed for lysis and immunoblotting as previously described.

### Cell migration and proliferation assays

For the cell migration assay, 48 h after transfection with control or UBA1 siRNA, HUVECs were seeded in starvation media (MCDB131) at 3×10^4^ cells per well in an 8 μm pore size Transwell filter inserted into a 24-well companion plate (BD Biosciences, Oxford, UK). MCDB131 containing 25 ng/ml VEGF-A was added to the lower chambers to set up a chemotactic gradient for cells to migrate towards. Cells were incubated for 24 h before being fixed and stained with 0.2% (w/v) crystal violet in 20% (v/v) methanol. Non-migrated cells were removed from the upper chamber. 3-5 random fields were imaged per Transwell filter.

For the cell proliferation assay, 48 h after transfection with control or UBA1 siRNA, HUVECs were seeded at 2×10^3^ cells per well in 96-well plates in complete growth media. After 24 h cells were serum starved in MCDB131 for 2 h prior to stimulation with 25 ng/ml VEGF-A for 24 h. At the 20 h time point, 10 μM bromodeoxyuridine (BrdU) was added and a cell proliferation ELISA performed according to manufacturer's instructions (Roche Diagnostics, Burgess Hill, UK). Color change was developed using 3,3′5,5′-tetramethylbenzidine solution and the reaction quenched with 1 M H_2_SO_4_. Absorbance was measured at 450 nm using a variable wavelength 96-well Tecan Sunrise plate reader (Mannedorf, Switzerland).

### Endothelial tubulogenesis and monolayer wound assays

For the tubulogenesis assay, HUVECs transfected with siRNA were seeded onto a bed of confluent primary human fibroblasts and stimulated with 25 ng/ml VEGF-A every 48 h for 7 days. Co-cultures were grown in 50:50 ECGM and DMEM [with 10% (v/v) FCS, 1% sodium pyruvate, 1% non-essential amino acids]. Tubules were stained with endothelial-specific marker, PECAM-1, overnight and incubated in anti-mouse secondary antibody (Alexa Fluor 594) and DNA-binding dye, DAPI, for 2 h at room temperature. Images were acquired using an Evos-fI inverted digital microscope. Five random fields were imaged per well at 10× magnification at room temperature. Both total tubule length and the number of branch points were quantified from each photographic field using the open source software AngioQuant (www.cs.tut.fi/sgn/csb/angioquant) and values averaged.

For the monolayer wound assay, endothelial cells were transfected with control or UBA1 siRNA and grown to a confluent monolayer in ECGM for 48 h. The cell monolayer was scratched using a 1 ml blue plastic pipette tip at the 0 h time point and stimulated with 25 ng/ml VEGF-A. After 24 h, wound closure was captured using an Evos-fI inverted digital microscope and scratch width quantified using ImageJ.

### Statistical analysis

This was performed using a one-way analysis of variance (ANOVA) and Tukey's post-test analysis for multiple comparisons or two-way ANOVA followed by the Bonferroni multiple comparison test using GraphPad Prism software (La Jolla, USA). Significant differences between control and test groups were evaluated with *P<0.05, **P<0.01, ***P<0.001 and ****P<0.0001 indicated on the graphs. Error bars in graphs denote mean±s.e.m. of results from at least three independent experiments.
